# Implementation of risk-based lipid-lowering therapies in older (age ≥ 65 years) and very-old adults (age ≥ 75 years) with ischemic heart disease in the greater Salzburg region

**DOI:** 10.3389/fphar.2024.1357334

**Published:** 2024-06-19

**Authors:** Kristen Kopp, Lukas J. Motloch, Bernhard Wernly, Alexander E. Berezin, Victoria Maringgele, Anna Dieplinger, Uta C. Hoppe, Michael Lichtenauer

**Affiliations:** ^1^ Department of Internal Medicine II, Division of Cardiology, Paracelsus Medical University, Salzburg, Austria; ^2^ Department of Internal Medicine II, Salzkammergut Klinikum, OOEG, Voecklabruck, Austria; ^3^ Department of Cardiology, Kepler University Hospital, Medical Faculty, Johannes Kepler University, Linz, Austria; ^4^ Institute for General-, Family- and Preventive Medicine, Paracelsus Medical University, Salzburg, Austria; ^5^ Department of Psychiatry, Psychotherapy and Psychosomatics, Paracelsus Medical University, Salzburg, Austria; ^6^ Institute for Nursing and Practice, Paracelsus Medical University, Salzburg, Austria

**Keywords:** older adults, low-density lipoprotein cholesterol, lipid-lowering therapy, guidelines, ST-segment elevation myocardial infarction, very-high risk

## Abstract

**Introduction:** European guidelines recommend the implementation of lipid-lowering therapies (LLTs) in adults (≥ 65 years) with established atherosclerotic cardiovascular disease (ASCVD) and for risk-based primary prevention in older adults (≤ 75 years), yet their use in very-old adults (> 75 years) is controversial, discretionary, and oriented on the presence of risk factors. The aim of this retrospective study is to assess guideline-directed LLT implementation and low-density lipoprotein cholesterol (LDL-C) target achievement in high-/very-high-risk older/very-old adults (65–74 and ≥ 75 years) at presentation for ST-segment elevation myocardial infarction (STEMI) and also to assess evidence-based care delivery to older adults in our region.

**Methods:** All STEMI patients with available LDL-C and total cholesterol presenting for treatment at a large tertiary center in Salzburg, Austria, 2018–2020, were screened (*n* = 910). High-risk/very-high-risk patients (*n* = 369) were classified according to European guidelines criteria and divided into cohorts by age: < 65 years (*n* = 152), 65–74 years (*n* = 104), and ≥ 75 years (*n* = 113).

**Results:** Despite being at high-/very-high-risk, prior LLT use was < 40% in the total cohort, with no significant difference by age. Statin monotherapy predominated; 20%–23% of older/very-old adults in the entire cohort were using low-/moderate-intensity stains, 11%–13% were using high-intensity statins, 4% were on ezetimibe therapy, and none were taking proprotein convertase subtilisin/kexin type 9 (PCSK9) inhibitors. In the secondary prevention cohort, 53% of older/very-old patients used prior LLTs. Significantly higher percentages of older/oldest ASCVD patients (43% and 49%) met LDL-C targets < 70 mg/dL compared to patients < 65 years (29%; *p* = 0.033), although just 22% and 30% of these older groups attained stricter LDL-C targets of < 55 mg/dL. Low LLT uptake (16%) among older adults aged 64–74 years for primary prevention resulted in 17% and 10% attainment of risk-based LDL-C targets < 70 mg/dL and < 55 mg/dL, respectively. Oldest adults (≥ 75 years) in both primary and secondary prevention groups more often met risk-based targets than older and younger adults, despite predominantly receiving low-/moderate-intensity statin monotherapy.

**Conclusion:** Secondary prevention was sub-optimal in our region. Less than half of older/very-old adults with established ASCVD met LDL-C targets at the time of STEMI, suggesting severe care-delivery deficits in LLT implementation. Shortcomings in initiation of risk-based LLTs were also observed among high-/very-high-risk primary prevention patients < 75 years, with the achievement of risk-based LDL-C targets in 10%–48% of these patients.

## 1 Introduction

Cardiovascular diseases (CVDs) are the leading cause of morbidity and mortality worldwide and in Europe, claiming some 1.8 million lives in the European Union annually (Timmis et al., 2020), with ischemic heart disease followed by stroke as the most prevalent CVD condition (Vaduganathan et al., 2022). CVD poses a major burden not only to the individual patient but also to health systems, being the highest healthcare cost component in the European Union. It accounts for 11% of EU health expenditure and an estimated €282 billion in annual costs (Luengo-Fernandez et al., 2023), thus making prevention to reduce CVD risk an essential health policy priority. Atherothrombotic coronary artery disease (ASCVD) is a root cause of type I myocardial infarction (Thygesen et al., 2018). Underlying the development of ASCVD is the retention of low-density lipoprotein cholesterol (LDL-C) and other cholesterol-rich apolipoprotein B-containing lipoproteins within the artery walls (Ference et al., 2017). As well-described in the literature, increased LDL-C values are causally linked to ASCVD development, and inversely, lower LDL-C values are correlated with a lower risk of future adverse cardiovascular (CV) events (Boekholdt et al., 2014; Ference et al., 2017). Every 1 mmol/L or 38.7 mg/dL absolute reduction in LDL-C corresponds to approximately a 10% reduction in all-cause mortality and an estimated 21% reduction in the occurrence of major adverse vascular events (Cholesterol Treatment Trialists CTT Collaboration et al., 2010).

Age is a relevant factor for CVD development, and increasing age is associated with higher rates of adverse CVD events (Stoll et al., 2020). Age is also considered a primary driver of risk, as age equals cumulative exposure time to risk factors (Mach et al., 2020). According to a 2022 American Heart Association publication, older adults ≥ 75 years in the US are disproportionately affected by ischemic heart disease and account for 30%–40% of all hospitalized patients with acute coronary syndrome (ACS) and the majority of ACS deaths (Damluji et al., 2023). Although the last decade has witnessed a decline in CVD death rates in developed countries due to better prevention and treatment, a paradoxical increase in CVD burden in older adults is expected due to the demographic shift and expansion of populations aged 65 years and older, increased life expectancy, and larger populations of older adults with a history of CVD taking optimal therapies (Dai et al., 2016).

A large body of evidence has shown that use of statins and cholesterol absorption inhibitors such as ezetimibe produces significant reductions in vascular events in patients with established ASCVD across all age groups, as well as in primary prevention in older adults ≤ 75 with high-/very-high-risk CVD profiles (Catapano et al., 2016; Ouchi et al., 2019; Mach et al., 2020; Lettino et al., 2022). The European Society of Cardiology (ESC) and the European Atherosclerosis Society (EAS) in their jointly issued *Guidelines for the Management of Dyslipidemias* in 2016, and upgraded in 2019, thus recommend first-line treatment with statins for people aged > 65 years with established ASCVD in the same way as for younger patients to achieve risk-based LDL-C targets (Catapano et al., 2016; Mach et al., 2020), with the introduction of ezetimibe recommended if LDL-C targets remain unmet on the maximally tolerated statin dose (Mach et al., 2020). Newer classes of drugs such as proprotein convertase subtilisin/kexin type 9 inhibitors (PCSK9is) may be considered in primary prevention and recommended in secondary prevention if targets are not achieved despite the use of statin–ezetimibe combination therapies, although data on their use in older adults are limited (Mach et al., 2020; Nanna et al., 2023). While the 2016 and 2019 ESC/EAS *Guidelines* provided a scoring system (Systematic Coronary Risk Estimation, SCORE) to calculate the 10-year cumulative risk of a fatal CVD event, a new SCORE-OP (older persons) published in 2021 now supports clinicians for implementation of LLT in older adults ≥70 years in primary prevention (SCORE2-OP working group and ESC Cardiovascular risk collaboration et al., 2021). However, certain patient groups are identified as high-/very-high risk without the need for risk calculation and thus are targeted for LDL-C-lowering and lifestyle interventions (Catapano et al., 2016; Mach et al., 2020). Guideline-recommended LDL-C target levels are based on total individual CV risk with the necessary follow-up evaluation of treatment responses, as responses vary according to the individual (Boekholdt et al., 2014; Corn et al., 2023).

However, LLT use in very-old adults > 75 years is contentious, especially in primary prevention in patients without ASCVD or modifiable CVD risk factors, in part due to less robust data in this age group but also due to other considerations such as multi-morbidity, frailty, cognitive impairment, polypharmacy, impaired renal function, safety (prevented outcomes versus side effects), quality of life, and longevity (Lettino et al., 2022).

The concept of time to benefit (TTB) versus time to harm (TTH) has emerged with respect to implementation of preventative LLTs and prioritization of multiple therapies in older and multi-morbid individuals (Holmes et al., 2013). Some authors have reconsidered the appropriateness of statin prescription in older individuals, arguing that the benefit of statin treatment should guide clinical decisions and citing the Cholesterol Treatment Trialists Collaboration meta-analysis showing that a standard reduction of cholesterol in patients age > 75 years would lead to an absolute risk reduction of 0.6% per year with a resultant number needed to treat (NNT) of 167 to prevent one vascular event per year of therapy (Cholesterol Treatment Trialists CTT Collaboration et al., 2010; Ruscica et al., 2018). Meta-analyses of several large primary prevention lipid trials (ASCOT-LLA, JUPITER, HOPE, and CARDS trials) describe NNTs ranging from 21 to 62 to prevent the occurrence of one adverse CV event that may include nonfatal MI, stroke, or CV death with use of atorvastatin or rosuvastatin in older individuals (> 60, 65, or ≥ 70 years) (Ruscica et al., 2018) A recent meta-analysis of LDL-lowering in 244,090 patients published in the *Lancet* in 2020, however, found an unequivocal reduction in the risk of vascular events with both statin and non-statin LDL-C lowering treatments, reducing the incidence of the endpoints CV death, myocardial infarction, stroke, and coronary revascularization both in patients ≥75 years and <75 years and in primary as well as secondary prevention (Gencer et al., 2020).

The goal of primary and secondary prevention in older as well as younger patients is to prevent or delay the progression of ASCVD with manifestations such as myocardial infarction, stroke, critical limb ischemia, or CV death. Prevention of events potentially results not only in increased longevity and maintenance of functional status but also an improved quality of life for patients, in addition to potential reduction in healthcare system burden and costs.

### 1.1 Study aims

The aims of the study are 1) to assess the use of risk-based, guideline-recommended LLT among older adults aged 65–74 years and very-old adults ≥75 years with and without medical history of ASCVD at the time of presentation for STEMI in our region, 2) to contribute knowledge about the use of statin-based treatments, especially in the oldest adults ≥ 75 years as described in “Gaps in the Evidence” in the ESC/EAS Guidelines for the Management of Dyslipidemias, and 3) to determine risk-based LDL-C target achievement in a real-world STEMI population with a focus on older and very-old adults meeting high- and very-high-risk criteria.

## 2 Methods

### 2.1 Study design

All patients (*n* = 964) presenting with ST-segment elevation myocardial infarction (STEMI) between 1 January 2018 and 31 December 2020 at a single, large tertiary care center in Salzburg, Austria, were screened for this retrospective study. Our center functions as the primary 24/7 regional cardiac care provider, providing cardiac catheterization services to patients from the State of Salzburg (2023 population: 568,000) as well as the greater region, including parts of the States of Upper Austria, Styria, Tirol (Austria), and Bavaria, Germany.

The inclusion criteria are as follows: STEMI patients aged ≥ 18, with available LDL-cholesterol (LDL-C, mg/dL) and total cholesterol (TC, mg/dL) values drawn during baseline hospitalization for STEMI (*n* = 910). Patients (*n* = 54) without available LDL-C and/or TC values were excluded.

**FIGURE 1:**
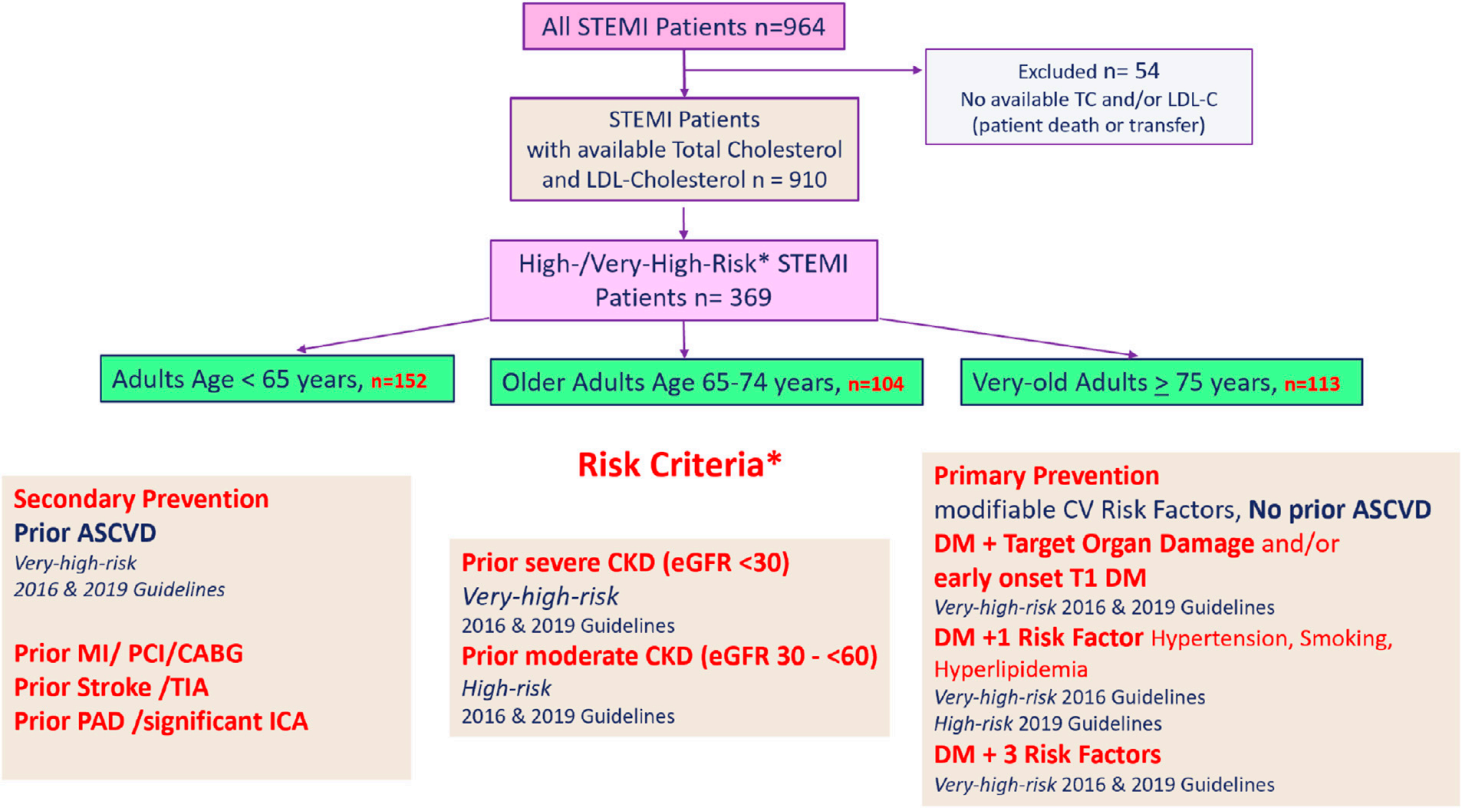
Patient inclusion and cohort stratification. STEMI, ST-segment elevation myocardial infarction; TC, total cholesterol mg/dL; LDL-C, low-density lipoprotein cholesterol; MI, myocardial infarction; PCI, percutaneous coronary intervention; CABG, coronary artery bypass graft; TIA, transient ischemic attack; PAD, peripheral arterial disease; ICA, internal carotid artery stenosis; CKD, chronic kidney disease; DM, diabetes mellitus; T1 DM, type I diabetes mellitus.

Patients with available LDL-C and TC values (*n* = 910) were then screened for the presence of high-/very-high-risk criteria, as described in the 2016 and the 2019 *ESC/EAS Guidelines for the Management of Dyslipidemias*, the current guidelines during the time of enrollment. A total of 324 patients met ESC/EAS high-risk or very-high-risk criteria when they presented for STEMI. Patients were stratified by age, and a description of age classification is provided in Section 2.2. The achievement of guideline-recommended, risk-based LDL-C targets (2016 and 2019) was then analyzed in all age groups. For patient inclusion and cohort stratification, see Figure 1.

A sub-analysis of patients with and without prior ASCVD was performed to assess the differences in guideline recommendations for LLT use by age and medical history conditions. Additionally, as severe kidney disease (eGFR <30 mL/min/1.73 m^2^) may affect prescription, dosing, and uptake of LLT, and as impaired renal function is common in older and very-old adults, a sub-analysis of all high-risk/very-high-risk patients with and without severe CKD was done.

Prior LLT in use at the time of admission for STEMI was recorded for all patients. Current daily use of 40 or 80 mg of atorvastatin or 20 or 40 mg of rosuvastatin constituted high-intensity statin use, while moderate-/low-intensity statin use was defined as current daily use of lower doses of atorvastatin, < 40 mg/day; rosuvastatin, <20 mg/day; or use of any other statins/doses (simvastatin, pravastatin, and fluvastatin in our study). Ezetimibe use in combination with statins or alone, use of PCSK9 inhibitors, and use of any other lipid-lowering therapy were recorded, although fibrates were the only non-statin/non-ezetimibe LLT in use among STEMI patients in this study.

To determine the presence of high-risk or very-high-risk characteristics, medical history was collected for all patients (see detailed description in Section 2.3). Laboratory parameters drawn during baseline hospitalization for STEMI included TC, triglycerides, HDL-C, non-HDL-C, LDL-C, HbA1C, CRP, eGFR, and cardiac markers such as high-sensitivity troponin T (hs-cTnT) and creatinine kinase (CK).

### 2.2 Patient age classification

Age is considered a major risk factor for CVD, yet the age cutoffs described in the literature and international guidelines are arbitrary, and the term “older adult” has been applied to individuals aged > 65 years, > 70 years, and > 75 years. The current definition according to the United Nations for older adults is a person ≥ 65 years of age (United Nations, 2023), while the World Health Organization (WHO) defines them as ≥ 60 years of age (World Health Organization, 2017). The ESC/EAS 2016 guidelines loosely use the term “older adults” without specifically defining its meaning, although citing literature using the age cutoffs, 65, 70, and 75 years (Catapano et al., 2016). The revised 2019 ESC/EAS guidelines used more specific terminology and defined older people as those > 65 years old, here recommending statin use in older people with ASCVD in the same way as for younger patients (secondary prevention) (Mach et al., 2020). The 2019 guidelines also utilized an age cutoff of 75, recommending risk-based statin use in patients aged ≤ 75 years (1A recommendation) and consideration of their use in high-risk/very-high-risk patients > 75 years of age, although a IIb/B recommendation was given due to gaps in evidence. Based on cutoffs of 65 and 75 years, we selected the following age cutoffs and terminology for use in our study: adults < 65 years, older adults aged 65–74 years, and very-old adults ≥ 75 years.

### 2.3 Patient risk classification and risk-based LDL-C targets

The period under consideration in our study (2018–2020) witnessed a change in the guidelines, both with respect to risk classification and guideline-directed, risk-based LDL-C targets. Both the 2016 and 2019 ESC/EAS guidelines defined patients with overt, documented ASCVD, either clinical or unequivocal on imaging, as having a very-high 10-year risk of fatal CVD. These include patients with previous myocardial infarction (MI) and/or coronary revascularization, previous stroke, or transient ischemic attack (TIA) and those with peripheral arterial disease (PAD) or significant internal carotid artery stenosis as seen on imaging. Patients with prior severe chronic kidney disease (CKD) with eGFR < 30 min/mL/1.73 m^2^ are also classified as very-high risk in both guideline years (Catapano et al., 2016; Mach et al., 2020). Additionally, diabetes mellitus (DM) patients with evidence of target organ damage, defined as microalbuminuria, retinopathy, or neuropathy, and/or early onset Type I DM (>20 years), and/or DM II patients presenting with three major risk factors such as smoking, hypertension, and hyperlipidemia are considered very-high risk for a fatal CVD event in both guidelines (Catapano et al., 2016; Mach et al., 2020). No risk score calculation is needed for patients with one or more of these very-high-risk criteria, and these patients will always qualify for medical LLT and lifestyle intervention. Patients with medical history of any of the aforementioned criteria were thus classified as very-high risk in our study.

With respect to guideline-directed risk-based LDL-C targets for very-high-risk patients, the 2016 Guidelines set an LDL-C target of < 70 mg/dL, while the revised 2019 ESC/EAS guidelines were more stringent, reducing the target to <55 mg/dL and urging “the lower the better” prevention strategies. Hence, both LDL-C cut-offs have been analyzed in our study.

Regarding risk classification in other DM patient groups, DM II patients presenting with just one additional risk factor such as hyperlipidemia (HLP), hypertension (HTN), or smoking are described in the 2016 Guidelines as very-high risk; however, DM plus a single risk factor was down-classified to high risk only in the 2019 Guidelines (Catapano et al., 2016; Mach et al., 2020). We therefore also included patients with high-risk criteria in our analysis due to guideline revision. Patients with DM but without end-organ damage, patients with moderate CKD (eGFR 30–59 mL/min/1.73 m^2^), and patients with TC > 310 mg/dL are also considered high-risk in both guideline years and are included in our study population. The risk factor familial hypercholesterolemia (FH) was not captured in our database, and while FH patients with established ASCVD are included by default, those without may not be captured (see limitations in Section 4.6). The 2016 and 2019 ESC/EAS Guidelines recommended LDL-C targets of <100 mg/dL and <70 mg/dL, respectively, for high-risk patients (Catapano et al., 2016; Mach et al., 2020), and therefore, these cutoffs were included in our analysis.

### 2.4 Measurement of LDL-C

All laboratory parameters were analyzed at the University Institute for Medical-Chemical Laboratory Diagnostics at the University Clinic Salzburg at the time of admission for STEMI. Plasma LDL-C concentration was determined using a c702 module of the Roche Cobas^®^ 8000 Analyzer (Roche Diagnostics Mannheim, Germany) according to the current manufacturer’s instructions. LDL-C was calculated using the Friedewald formula when triglyceride levels were <275 mg/dL; otherwise, a direct method of measurement of LDL-particle numbers was applied. According to EASC/EAS guidelines, both calculated and direct measurements of LDL-C show good alignment Ference et al., 2017. However, it must be noted that the reliability of the Friedewald LDL-C calculation may be influenced by a non-fasting state. Additionally, plasma LDL-C and LDL particle concentrations can become discordant in patient populations with certain comorbidities such as diabetes or hypertriglyceridemia; thus, ESC/EAS guidelines recommend analyzing non-HDL-C (Catapano et al., 2016; Mach et al., 2020). Due to the presence of these comorbidities in many high-/very-high-risk patients and because fasting status could not be reliably determined upon admission for STEMI, non-HDL-C values were therefore provided for all patients.

### 2.5 Estimating the glomerular filtration rate

The CKD–EPI formula was used to estimate the glomerular filtration rate (eGFR, mL/min/1.73 m^2^) (Levey et al., 2009).

### 2.6 Statistical analyses

All analyses were descriptive, and the data were summarized by age groups. A Shapiro–Wilk test confirmed the unequal distribution of data. A chi-square test was thus applied for categorical variables, which are reported as numbers and percentages. A Wilcoxon rank-sum test was applied for continuous variables. Here, data are reported as the median and interquartile range (IQR). A *p*-value < 0.05 is considered statistically significant. Stata/BE 18.0 software was used for statistical analysis (StatCorp. 2023. *Stata Statistical Software: Release 18*. College Station, TX: StatCorp LLC, United States).

### 2.7 Data extraction

Data were extracted from STEMI hospitalization charts, and admission, discharge, and laboratory reports were found in the ORBIS electronic medical records system (Agfa Healthcare, version 08043301.04110DACHL) and the medical records archiving system (Krankengeschichten Archiv System, Uniklinikum Salzburg, Softworx by Andreas Schwab ™, 2008) of the University Clinic Salzburg, Austria, and entered pseudo-anonymously into an Excel database.

### 2.8 Ethics declaration

The Ethics Commission of the State of Salzburg, Austria, approved this study on 12 April 2021 (EK-Nr. 1038/2021) and determined that no patient informed consent was required due to the retrospective study design. The data were handled in accordance with the Declaration of Helsinki and according to Good Clinical Practice (ICH-GCP).

## 3 Results

### 3.1 High-/very-high-risk older adults (65–74 years) and very-old adults (≥ 75 years)

Table 1 illustrates patient characteristics for all high-risk/very-high-risk patients (*n* = 369) for the entire STEMI cohort by age. Women comprised 14%, 26%, and 41% of the < 65 year, 65–74 year, and ≥ 75 year populations, respectively (*p* < 0.001). With respect to behavioral risk factors, high active smoking rates were observed in younger adults aged <65 years (59%) and in older adults aged 65–74 years (24%), exceeding the 2019 Austrian national average of 21% and the EU average of 18.4% daily active smokers (OECD, 2021). A greater number of former smokers were observed among older adults (29%) and very-old adults (27%) (*p* < 0.001). The median BMI of each age cohort was 28, 27, and 26 (*p* < 0.001), thus meeting the WHO classification of overweight (BMI 25 to <30 kg/m^2^), with the upper quartile of patients aged < 65 years and aged 65–74 years meeting the classification of obesity (World Health Organization Fact, 2023).

**TABLE 1:** Patient characteristics.

	Age < 65	Age 65–74	Age ≥ 75	*p*-value
	*N* = 152	*N* = 104	*N* = 113	
Age	57 (52–61)	70 (67–72)	79 (77–83)	<0.001
Gender				<0.001
Women	14% (22)	26% (27)	41% (46)	
Men	86% (130)	74% (77)	59% (67)	
BMI	28 (26–31)	27 (25–31)	26 (23–29)	<0.001
Smoking				<0.001
Current	59% (90)	24% (25)	11% (12)	
Former	14% (21)	29% (30)	27% (30)	
Hypertension	77% (116)	82% (84)	90% (102)	0.023
Hypertension pretreated	52% (78)	67% (68)	77% (87)	<0.001
Hyperlipidemia	79% (119)	80% (82)	76% (86)	0.77
Hyperlipidemia pretreated	32% (48)	37% (38)	36% (41)	0.69
Prior MI	34% (51)	27% (28)	26% (29)	0.31
Prior PCI/CABG	42% (64)	38% (39)	30% (34)	0.13
Prior Stroke/TIA	7% (10)	9% (9)	19% (21)	0.006
Prior PAD/ICA	14% (22)	18% (19)	22% (25)	0.27
Prior renal insufficiency	11% (17)	29% (30)	50% (56)	<0.001
Diabetes mellitus	52% (79)	53% (55)	40% (45)	0.079
Diabetes pretreated	29% (43)	39% (40)	26% (29)	0.042
Prior heart failure	14% (21)	12% (12)	15% (17)	0.73
LVEF %	40 (35–50)	40 (35–50)	40 (35–50)	0.67
Atrial fibrillation	3% (4)	9% (9)	21% (24)	<0.001
Cancer				0.012
Active	2% (3)	7% (7)	4% (4)	
Previous	2% (3)	7% (7)	11% (12)	
Death during STEMI hospitalization	7% (11)	10% (10)	17% (19)	0.041

ASCVD, atherosclerotic coronary vascular disease; BMI, body mass index; MI, myocardial infarction; PCI, percutaneous coronary intervention; CABG, coronary artery bypass graft; TIA, transient ischemic attack; PAD, peripheral arterial disease; ICA, internal carotid artery stenosis; LVEF, left ventricular ejection fraction; STEMI, ST-segment elevation myocardial infarction.

Among classic CV risk factors, previously diagnosed hypertension was most common, occurring in 77%–90% of high-/very-high-risk STEMI patients, and was most prevalent in very-old adults aged ≥ 75 years (*p* < 0.001). Previously diagnosed hyperlipidemia was observed in 76%–80% of STEMI patients, without significant differences between age groups (*p* = 0.77). Note that while hypertension was pretreated in 52%–77% of patients, again most often in very-old adults ≥75 years (*p* < 0.001), pretreatment of hyperlipidemia was observed in just 32%–37% of STEMI patients with no significant differences between age groups (*p* = 0.69).

With respect to established ASCVD at presentation for STEMI, prior coronary artery disease was observed in 30%–42% of patients, with no significant differences between age groups; prior peripheral arterial disease (PAD) and/or internal carotid arterial disease were noted in 14%–22% of patients and were more common in older and very-old adults, although the differences between age groups were non-significant (*p* = 0.27). Patients with previous ischemic stroke or TIA comprised 7%–19% of the STEMI patient population, and this finding of ASCVD medical history was most common in older and very-old adults (*p* = 0.006).

Among other patient characteristics, medical history of CKD (eGFR <60 mL/min/1.73 m^2^) was observed in 29% of older adults 65–74 years and in 50% of very-old adults ≥ 75 years, compared with 11% in adults < 65 years (*p* < 0.001). Older patients also had significantly higher rates of atrial fibrillation and active/prior cancer than patients < 65 years, although prior heart failure and left ventricular ejection fraction (40%, IQR 35–50) did not differ significantly between age cohorts. The incidence of prior diabetes mellitus, although more common in patients <75 years (52%–52% vs. 40%), was not significantly different between age groups. Compared to just 7% (*n* = 6) of younger adults, 12% (*n* = 7) of older adults and 17% (*n* = 12) of very-old adults died during hospitalization for STEMI; however, the difference in incidence between age groups was not significant (*p* = 0.12).

Laboratory values are listed by age group in Table 2. In this study, the most notable findings are significant differences observed in lipid profiles and renal function (eGFR) between age groups. Younger patients < 65 years showed highest TC (176 mg/dL, IQR 148–208) compared to adults aged 65–74 and ≥ 75 years (157 mg/dL, IQR 130–194; 152 mg/dL, IQR 129–200; *p* = 0.004), as well as highest LDL-C (103 mg/dL, IQR 76–135) compared to older adults (86 mg/dL, IQR 64–118) and oldest adults (86 mg/dL, IQR 58–121), *p* = 0.004. Parallel to higher LDL-C values, non-HDL-C was also highest in patients < 65 years compared to older and very-old adults (134 mg/dL, IQR 100–166; 108 mg/dL, IQR 82–142; and 103 mg/dL, IQR 72–146, respectively; *p* < 0.001). Additionally, significantly more patients < 65 years had triglyceride levels in excess of 275 mg/dL (15%; *p* = 0.009) compared to older and very-old adults. Regarding other non-lipid parameters, while there were no significant differences between age groups for the parameters HbA1C (6%, *p* = 0.082) and CRP (3–4, *p* = 0.30), renal function (eGFR) was significantly reduced in the older and very-old adult population (80 mg/dL IQR 67–90 adults vs. 68 mg/dL IQR 50–82 older adults, 52, IQR 41–70 oldest adults; *n* = <0.001). A sub-analysis of TC and LDL-C values in patients who died versus those who survived baseline hospitalization showed no significant differences between groups: TC 162 mg/dL, IQR 128–210 vs 166 mg/dL, IQR 137–205; *p* = 0.76 and LDL-C: 81 md/dL, IQR 67–114 vs. 94 mg/dL, IQR 64–128;; *p* = 0.75.

**TABLE 2:** Laboratory parameters.

	Age < 65	Age 65–74	Age ≥ 75	*p*-value
	*N* = 152	*N* = 104	*N* = 113	
Cardiac markers				
hsTnT (ng/L, IQR)	3,204 (1,185–6,353)	3,470 (1,431–6,761)	3,813 (1,359–8,025)	0.62
CK (U/L, IQR)	1,445 (642–3,023)	1,281 (599–2,832)	1,213 (525–2,159)	0.099
Lipid parameters				
Total cholesterol (mg/dL, IQR)	176 (148–208)	157 (130–194)	152 (129–200)	0.004
Triglycerides (mg/dL, IQR)	144 (102–213)	108 (71–168)	105 (77–141)	<0.001
HDL (mg/dL, IQR)	42 (35–50)	45 (37–59)	50 (40–61)	<0.001
Non-HDL (mg/dL, IQR)	**134 (100–166)**	**108 (82–142)**	**103 (72–146)**	**<0.001**
LDL (mg/dL, IQR)	**103 (76–135)**	**86 (64–118)**	**82 (58–121)**	**0.004**
Other parameters				
HbA1C (%, IQR)	6 (6–8)	6 (6–7)	6 (6–7)	0.082
CRP	3 (1–10)	3 (1–13)	4 (2–11)	0.30
eGFR	80 (67–90)	68 (50–82)	52 (41–70)	<0.001
Non-HDL				0.006
Non-HDL <85 mg/dL	16% (22)	29% (29)	33% (32)	
Non-HDL 85–99 mg/dL	9% (12)	14% (14)	12% (12)	
Non-HDL >99 mg/dL	76% (106)	57% (58)	55% (54)	
Triglycerides				0.009
Triglycerides <275 mg/dL	85% (122)	92% (93)	96% (100)	
Triglycerides ≥275 mg/dL	15% (22)	8% (8)	4% (4)	
LDL				0.022
LDL <55 mg/dL	6% (9)	17% (17)	23% (23)	
LDL 55–69 mg/dL	16% (23)	15% (15)	16% (16)	
LDL 70–99 mg/dL	25% (36)	25% (25)	24% (24)	
LDL >99 mg/dL	52% (75)	43% (43)	38% (39)	

hsTnT, high-sensitive troponin T; HDL, high-density lipoprotein; non-HDL, non-high-density lipoprotein; LDL, low-density lipoprotein. The parameters highlighted in bold are those required by ESC/EAS to determine LDL-C and non-HDL target attainment.

With respect to the focus of our study, see Table 3 for the achievement of LDL-C guideline targets in older/very-old adults with high-/very-high-risk criteria at the time of presentation for STEMI.

**TABLE 3:** Prior lipid-lowering therapies and ESC/EAS^a^ lipid target achievement.

	Age < 65	Age 65–74	Age ≥ 75	*p*-value
	*N* = 152	*N* = 104	*N* = 113	
Hyperlipidemia pretreated	32% (48)	37% (38)	36% (41)	0.69
Statin intensity				0.90
Low/moderate-intensity+	16% (25)	23% (24)	20% (23)	
High-intensity*	13% (20)	11% (11)	13% (15)	
Intensity unknown	1% (2)	1% (1)	1% (1)	
Pretreatment with ezetimibe	5% (7)	4% (4)	4% (5)	0.45
Unknown	0% (0)	1% (1)	0% (0)	
Pretreatment with other LLT				0.47
Fibrate	1% (2)	1% (1)	0% (0)	
PCSK9i	0%	0%	0%	
Known statin intolerance	1% (2)	3% (3)	2% (2)	0.66
LLT target achievement				
Non-HDL				0.006
Non-HDL <85 mg/dL	16% (22)	29% (29)	33% (32)	
Non-HDL 85–99 mg/dL	9% (12)	14% (14)	12% (12)	
Non-HDL >99 mg/dL	76% (106)	57% (58)	55% (54)	
LDL				0.022
**LDL <55 mg/dL**	**6% (9)**	**17% (17)**	**23% (23)**	
**LDL 55–69 mg/dL**	**16% (23)**	**15% (15)**	**16% (16)**	
LDL 70–99 mg/dL	25% (36)	25% (25)	24% (24)	
LDL >99 mg/dL	52% (75)	43% (43)	38% (39)	

*High-intensity statins: atorvastatin ≥40 mg and rosuvastatin ≥20 mg.

^+^low/moderate-intensity statins: atorvastatin <20 mg, rosuvastatin <20 mg, or all other statins/doses such as simvastatin, pravastatin, and fluvastatin; LLT, lipid-lowering therapy; PCSK9i, proprotein convertase subtilisin/kexin type 9 inhibitors; LDL, low-density lipoprotein. The parameters highlighted in bold are those required by ESC/EAS to determine LDL-C and non-HDL target attainment.

^a^ ESC/EAS Guidelines, European Society of Cardiology (ESC)/European Atherosclerosis Society (EAS) Guidelines for the Management of Dyslipidemias (2016 and 2019).

During presentation for STEMI, 57% of older adults had an LDL-C < 100 mg/dL, the 2016 target for high-risk patients, yet just 32% of older adults had an LDL-C < 70 mg/dL, the guideline target for very-high-risk patients in 2016 and high-risk patients in 2019. Only 17% of older adults met the more stringent 2019 LDL-C target of <55 mg/dL for very-high-risk patients at the time of STEMI. With respect to secondary non-HDL targets as listed in the 2019 guidelines, 43% of older adults met high risk and just 29% met very-high-risk non-HDL guideline targets at presentation for STEMI.

Among the oldest adults aged ≥ 75 years at the time of STEMI presentation, 63% met the 2016 LDL-C < 100 mg/dL for high-risk patients, while just 39% achieved the LDL-C target of < 70 mg/dL for high-/very-high-risk patients according to the 2016 and 2019 guidelines, respectively. Approximately 23% of very-old adults met the stricter 2019 LDL-C very-high-risk goal of <55 mg/dL. Regarding secondary non-HDL goals, 45% met high-risk and 33% met very-high-risk targets. Note that patients < 65 years had the lowest achievement of guideline targets, with just 22% having LDL-C <70 mg/dL and only 6% meeting LDL-C <55 mg/dL targets at the time of presentation for STEMI, significantly lower than achievement among older patient groups (*p* = 0.022). Aligning with these observations, just 25% met non-HDL secondary targets <100 mg/dL for high-risk patients and just 16% met the stringent targets for very-high-risk patients, both significantly lower than those of the older and oldest adult populations (*p* = 0.006).

Hyperlipidemia was pretreated in just 32%–36% of our high-risk/very-high-risk STEMI population, with no significant differences between age groups (*n* = 0.69) (refer to Table 3 for LLT implementation). Low-/moderate-intensity statins were the most commonly prescribed LLTs, taken by 16%, 23%, and 20% (*p* = 0.9) adults, older adults, and very-old adults, respectively, at the time of presentation for STEMI, but without significant differences between age groups. Just 13%, 11%, and 13% of STEMI patients in each age category were treated with high-intensity statins, and only few (5%–4%) were treated with ezetimibe, either in combination with statin therapy or alone, also without significant differences between age groups (*p* = 0.45). Isolated patients were taking fibrates at the time of STEMI. No patient in the entire STEMI cohort was treated with PCSK9is. Known statin intolerance was low and ranged from 1% in adults < 65 years to 3%–2% in older and very-old adults (*p* = 0.66).

### 3.2 Very-high-risk patients with previously established ASCVD

Table 4 shows the characteristics of patients with and without previously established ASCVD. Regarding patients with previously diagnosed ASCVD (see Table 4, left column), these secondary prevention patients are always considered very-high risk and thus require intensive lipid-lowering therapy to meet risk-based LDL-C targets as well as lifestyle interventions for risk reduction. The majority of patients in each age group had prior ASCVD at presentation for STEMI (58.5% of patients < 65 years, *n* = 89; 56.7% of older adults 65–74 years, *n* = 59; and 61.9% of very-old adults ≥ 75 years, *n* = 70). Women represented 27% of the older adult age group and 40% of the very-old adult STEMI population with established ASCVD at the time of admission. Among modifiable risk factors, at least half of the patients in each age group were classified as overweight, with the upper quartile of adults and older adults < 75 years meeting the classification of obesity. The highest rates of active smoking were observed among adults < 65 (63%) and older adults < 75 years (32%).

**TABLE 4:** Patient characteristics (patients with and without prior ASCVD).

Patients with prior ASCVD					Patients without prior ASCVD				
	Age <65	Age 65–74	Age ≥75	*p*-value		Age <65	Age 65–74	Age ≥75	*p*-value
	*N* = 89	*N* = 59	*N* = 70			*N* = 63	*N* = 45	*N* = 43	
Age	57 (51–61)	70 (66–73)	80 (77–83)	<0.001	Age	58 (55–61)	70 (68–72)	79 (77–83)	<0.001
Gender				<0.001	Gender				0.019
Women	12% (11)	27% (16)	40% (28)		Women	17% (11)	24% (11)	42% (18)	
Men	88% (78)	73% (43)	60% (42)		Men	83% (52)	76% (34)	58% (25)	
BMI	28 (25–31)	27 (24–31)	26 (24–29)	0.19	BMI	28 (27–32)	28 (26–32)	26 (23–29)	<0.001
Smoking				<0.001	Smoking				<0.001
Current	63% (56)	32% (19)	10% (7)		Current	54% (34)	13% (6)	12% (5)	
Former	16% (14)	24% (14)	31% (22)		Former	11% (7)	36% (16)	19% (8)	
Hypertension	76% (67)	81% (48)	91% (64)	0.041	Hypertension	79% (49)	82% (36)	88% (38)	0.46
Hypertension pretreated	52% (45)	71% (42)	79% (55)	0.001	Hypertension pretreated	70% (43)	66% (29)	65% (28)	0.084
Hyperlipidemia	85% (76)	90% (53)	83% (58)	0.52	Hyperlipidemia	70% (43)	66% (29)	65% (28)	0.81
Hyperlipidemia pre-treated	45% (39)	53% (31)	53% (37)	0.52	Hyperlipidemia pre-treated	15% (9)	16% (7)	9% (4)	0.63
Prior MI	57% (51)	47% (28)	41% (29)	0.13	Prior MI	0% (0)	0% (0)	0% (0)	
Prior PCI/CABG	72% (64)	66% (39)	49% (34)	0.009	Prior PCI/CABG	0% (0)	0% (0)	0% (0)	
Prior Stroke/TIA	11% (10)	15% (9)	30% (21)	0.008	Prior Stroke/TIA	0% (0)	0% (0)	0% (0)	
Prior PAD/ICA	25% (22)	32% (19)	36% (25)	0.30	Prior PAD/ICA	0% (0)	0% (0)	0% (0)	
Prior renal insufficiency	8% (7)	27% (16)	37% (26)	<0.001	Prior renal insufficiency	16% (10)	31% (14)	70% (30)	<0.001
Diabetes mellitus	23% (20)	36% (21)	34% (24)	0.16	Diabetes mellitus	94% (59)	76% (34)	49% (21)	<0.001
Diabetes pre-treated	13% (13)	29% (17)	20% (14)	0.29	Diabetes pretreated	49% (31)	51% (23)	35% (15)	0.048
Prior heart failure	23% (20)	15% (9)	16% (11)	0.39	Prior heart failure	2% (1)	7% (3)	14% (6)	0.042
LVEF %	40 (35–50)	42 (35–50)	40 (35–50)	0.99	LVEF %	40 (35–50)	40 (35–50)	40 (35–47)	0.33
Atrial fibrillation	3% (3)	8% (5)	27% (19)	<0.001	Atrial fibrillation	2% (1)	9% (4)	12% (5)	0.095
Cancer				0.039	Cancer				0.50
Active	2% (2)	8% (5)	14% (10)		Active	2% (1)	4% (2)	5% (2)	
Previous	2% (2)	7% (4)	4% (3)		Previous	2% (1)	7% (3)	2% (1)	
Death during hospitalization	7% (6)	12% (7)	17% (12)	0.12	Death during hospitalization	8% (5)	7% (3)	16% (7)	0.25

ASCVD, atherosclerotic coronary vascular disease; BMI, body mass index; MI, myocardial infarction; PCI, percutaneous coronary intervention; CABG, coronary artery bypass graft; TIA, transient ischemic attack; PAD, peripheral arterial disease; ICA, internal carotid artery stenosis; LVEF, left ventricular ejection fraction; STEMI, ST-segment elevation myocardial infarction.

Regarding ASCVD qualifying conditions, previous incidence of myocardial infarction was observed in 57% of patients < 65 years and 47% and 41% of older and very-old adults, respectively, with no significant differences by age (*p* = 0.13). Prior coronary revascularization was significantly more prevalent in younger patients (72% versus 66% of older adults and 49% of very-old adults, *p* = 0.009). Prevalence of prior PAD/ICA did not significantly differ between age groups, although it was more common in older and very-old adults (*p* = 0.30). With respect to the prevalence of prior stroke or TIA, no significant differences were observed between age groups, although this medical history was more common in older (32%) and very-old adults (36%) than in adults < 65 years (25%, *p* = 0.3).

Older and very-old ASCVD patients more commonly had a medical history of hypertension compared to patients < 65 years (81% and 91% versus 76%, *p* = 0.041). The prevalence of treatment for hypertension also increased significantly with age, with just 52% of patients < 65 years on treatment for hypertension at presentation for STEMI compared to 71% among older adults and 79% (*p* = 0.001) of very-old adults. Equally common was the presence of hyperlipidemia in patients with prior ASCVD, yet there were no significant differences between age groups. While previous hyperlipidemia was observed in 85% of adults, 90% of older adults, and 83% of very-old adults (*p* = 0.52), only 45%–53% of very-high risk ASCVD patients were actually on treatment at the time of STEMI, with no significant differences observed between age groups (patients < 65 years, 45% versus older adults (53%) and very-old adults (53%); *p* = 0.52).

There were significant differences between age groups with respect to the occurrence of severe and moderate chronic kidney disease, an important CV risk factor, which was significantly more prevalent in older and oldest adults (27% and 37%, respectively) compared with adults < 65 years (8%, *p* < 0.001). While there were no significant differences in the prevalence of diabetes mellitus between age groups, older adults and very-old adults were more often previously diagnosed compared to younger patients (36% and 34% versus 23% respectively, *p* = 0.16). Furthermore, prevalence of prior and active cancer (*p* < 0.001) and atrial fibrillation (*p* < 0.001) was more common in older and very-old adults. Prior heart failure, in contrast, was more prevalent in younger patients aged < 65 years (23%) compared to its prevalence in 15%–16% of older and very-old adults, but this was not significant (*p* = 0.39). Death during STEMI hospitalization occurred in 7%–17% of patients, and although more common in older and oldest adults, the difference between age groups was non-significant.

### 3.3 LLTs and LDL-C target achievement in high-risk patients with prior ASCVD

The median LDL-C for this very-high-risk ASCVD population was 97 mg/dL (IQR 64, 135) in younger adults and significantly lower in older adults (77 mg/dL, IQR 58, 108) and very-old adults (77 mg/dL, IQR 51, 109) (*p* = 0.004). TC was 172 mg/dL (IQR 140, 210), 151 mg/dL (127, 178), and 142 mg/dL (122, 186) in younger, older, and very-old adults, respectively (*p* = 0.005). Regarding the achievement of risk-based lipid targets, see Table 5.

**TABLE 5:** Lipid-lowering therapies and ESC/EAS^a^ lipid target achievement in patients with and without prior ASCVD.

Patients with prior ASCVD					Patients without prior ASCVD				
	Age <65	Age 65–74	Age ≥75	*p*-value		Age <65	Age 65–74	Age ≥75	*p*-value
	*N* = 89	*N* = 59	*N* = 70			*N* = 63	*N* = 45	*N* = 43	
Hyperlipidemia pretreated	45% (39)	53% (31)	53% (37)	0.52	Hyperlipidemia pretreated	15% (9)	16% (7)	9% (4)	0.63
Statin intensity				0.66	Statin intensity				0.84
Low/moderate-intensity+	21% (19)	34% (20)	29% (20)		Low/moderate-intensity+	10% (6)	9% (4)	7% (3)	
High-intensity*	20% (18)	17% (10)	20% (14)		High-intensity*	3% (2)	2% (1)	2% (1)	
Intensity unknown	2% (2)	0% (0)	1% (1)		Intensity unknown	0% (0)	2% (1)	0% (0)	
Pretreatment with ezetimibe	8% (7)	5% (3)	7% (5)	0.54	Pretreatment with ezetimibe	0% (0)	2% (1)	0% (0)	0.42
Unknown	0% (0)	0% (0)	0% (0)		Unknown	0% (0)	2% (1)	0% (0)	
Pre-treatment with other LLT				0.55	Pre-treatment with other LLT				0.62
Fibrate	1% (1)	0% (0)	0% (0)		Fibrate	2% (1)	2% (1)	0% (0)	
PCSK9i	0% (0)	0% (0)	0% (0)		PCSK9i	0% (0)	0% (0)	0% (0)	
Known statin intolerance	2% (2)	3% (2)	3% (2)	0.92	Known statin intolerance	0% (0)	2% (1)	0% (0)	0.31
LLT target achievement^a^					LLT target achievement^a^				
Non-HDL				0.028	Non-HDL				0.18
Non-HDL <85 mg/dL	20% (17)	36% (21)	42% (25)		Non-HDL <85 mg/dL	9% (5)	19% (8)	18% (7)	
Non-HDL 85–99 mg/dL	10% (8)	16% (9)	8% (5)		Non-HDL 85–99 mg/dL	7% (4)	12% (5)	18% (7)	
Non-HDL >99 mg/dL	70% (58)	48% (28)	50% (30)		Non-HDL >99 mg/dL	84% (48)	70% (30)	63% (24)	
LDL				0.033	LDL				0.90
LDL <55 mg/dL	7% (6)	22% (13)	30% (19)		LDL <55 mg/dL	5% (3)	10% (4)	10% (4)	
LDL 55–69 mg/dL	22% (18)	21% (12)	19% (12)		LDL 55–69 mg/dL	8% (5)	7% (3)	10% (4)	
LDL 70–99 mg/dL	25% (21)	21% (12)	21% (13)		LDL 70–99 mg/dL	25% (15)	31% (13)	28% (11)	
LDL >99 mg/dL	46% (38)	36% (21)	30% (19)		LDL >99 mg/dL	62% (37)	52% (22)	51% (20)	

^+^low/moderate-intensity statins: atorvastatin < 20 mg, rosuvastatin < 20 mg, or all other statins/doses such as simvastatin, pravastatin, and fluvastatin; LLT, lipid-lowering therapy; PCSK9i, proprotein convertase subtilisin/kexin type 9 inhibitor; non-HDL, Non-high-density lipoprotein; LDL, low-density lipoprotein.

*High-intensity statins: atorvastatin ≥40 mg and rosuvastatin ≥20 mg.

^a^ ESC/EAS Guidelines, European Society of Cardiology (ESC)/European Atherosclerosis Society (EAS) Guidelines for the Management of Dyslipidemias (2016 and 2019).

Less than half of older and very-old ASCVD patients met the 2016 guideline target LDL-C <70 mg/dL for very-high-risk patients (43% of older adults and 49% of very-old adults), with even fewer achieving the more stringent 2019 LDL-C target <55 mg/dL at the time of presentation of STEMI (22% of older adults and 30% of very-old adults). To be mentioned, significantly lower percentages of younger adults < 65 years achieved the 2016 (29%) and 2019 LDL-C targets (7%), respectively, at the time of admission for STEMI (*p* = 0.033). The achievement of guideline-directed non-HDL secondary targets paralleled findings for LDL-C, with 42% and 36% of older adults meeting 2019 high- and very-high-risk non-HDL targets, respectively, and 50% and 42% of oldest adults ≥ 75 years meeting high-/very-high risk non-HDL secondary targets, respectively.

Fifty-three percent of both older adults 65–74 years and oldest adults ≥ 75 years with prior ASCVD were on treatment for hyperlipidemia at the time of STEMI presentation, primarily statin monotherapy. Low- or moderate-intensity statin therapy predominated, used in 34% of older adults, 29% of very-old adults, and 21% of younger adults, with no significant differences between age groups (*p* = 0.66). Only 17% of older adults and 20% of the oldest adults were taking high-intensity statins at the time of STEMI presentation. Pre-treatment with ezetimibe was uncommon, with 8% of younger adults, 5% of older adults, and 7% of very-old adults taking ezetimibe either in combination with statins or as monotherapy. None of our ASCVD patients had been given PCSK9 inhibitors. Just 2%–3% of ASCVD patients had documented statin intolerance.

### 3.4 High-risk/very-high-risk patients without previously diagnosed ASCVD

For characteristics of patients without previously established ASCVD at the time of STEMI presentation, see Table 4, right column. Approximately 43.3% of older adults and 38.1% of very-old adults did not have a medical history of ASCVD at the time of STEMI presentation, yet other risk factors qualified them as high /very-high risk with a need for primary prevention treatment. Women represented 24% of older adults and 42% of very-old adults, yet 17% of the younger adult population (*p* = 0.019). Median BMI was 28 in younger and older adults and 26 in the oldest adults, meeting the criteria for overweight, with the upper quartile of younger and older adults fulfilling the classification of obesity. In contrast, the lower quartile of very-old adults had a normal weight (*p* < 0.001). Significantly more younger adults <65 years were active smokers (54%) compared with older adults (13%) and very-old adults (12%) (*p* < 0.001), although there were higher rates of previous smoking among older (36%) and very-old adults (19%). Again, hypertension was a common comorbidity in this population, observed in 79% of young adults, 82% of older adults, and 88% of very-old adults. Hypertension was pretreated in 70%, 66%, and 65% of younger, older, and very-old adults, respectively, without significant differences by age (*p* = 0.084). While hyperlipidemia was a common medical history finding in 70% of younger adults <65 years, 66% of older adults aged 65–74, and 65% of oldest adults ≥75 years, pretreatment was observed in just 15% 16%, and 9% of these high-risk/very-high-risk patients, respectively, with no significant differences between age groups (*p* = 0.63).

The most common high- or very-high-risk criteria for 10-year fatal CVD among the patient population without previously established ASCVD differed significantly by age group. Diabetes with one or more additional risk factors such as hypertension, hyperlipidemia, and/or smoking was common in 94% of younger adults < 65 years and in 76% of older adults aged 65–74 years, yet in just 49% of very-old adults (*p* < 0.001). Prior renal insufficiency, in contrast, was observed in just 16% of younger adults, 31% of older adults, and 70% of very -old adults (*p* < 0.001). In addition, very-old adults more commonly had prior heart failure (14%) compared to older adults (7%) and younger adults (2%) (*p* = 0.042). In addition, the presence of atrial fibrillation (9% and 12%, respectively) as well as a medical history of active (4%–5%) or previous (7%, 2%) cancer were more common in older and very-old adults, without significant differences between age groups. Death during hospitalization for STEMI among patients without established ASCVD did not differ significantly between age groups, although it more commonly occurred in very-old adults (17%) compared with older adults (7%) and younger adults (8%) (*p* = 0.25).

### 3.5 LLT and LDL-C target achievement in high-risk patients without prior ASCVD

LDL-C in high-/very-high-risk patients presenting without known ASCVD was 108 mg/dL (86, 134) in younger adults, 104 mg/dL in older adults (76, 132), and 101 mg/dL (70, 147) in very-old adults (*p* = 0.58). TC was 182 (162, 206), 171 mg/dL (148, 215), and 175 mg/dL (142, 214) in these three groups, respectively (*p* = 0.58). Regarding the achievement of risk-based lipid targets among patients without prior ASCVD upon admission for STEMI, see Table 5, right column.

While less than half of older and very-old adults (48%) met the 2016 high-risk target LDL-C < 100 mg/dL, less than 20% of older and very-old patients in this population met the 2016 very-high-risk guideline target LDL-C <70 mg/dL (17% of older adults and 20% of very-old adults), with just 10% of older and very-old adults achieving the stricter 2019 LDL-C target <55 mg/dL at the time of STEMI presentation. Note that LDL-C target achievement was lowest among younger adults < 65 years, with 13% meeting 2016 LDL-C < 70 mg/dL and 5% meeting 2019 LDL-C target < 55 mg/dL at the time of admission for STEMI, though differences by age were not significant (*p* = 0.9). The achievement of guideline-directed non-HDL secondary targets was accordingly low, with just 31% and 19% of older adults meeting 2019 high- and very-high-risk non-HDL targets, respectively, and 36% and 18% of oldest adults ≥ 75 years meeting high- and very-high-risk non-HDL secondary targets, respectively.

Pretreatment for hyperlipidemia at the time of STEMI presentation was lowest in this high-risk/very-high-risk cohort of patients without previously established ASCVD at admission for STEMI. Only 16% of older adults 65–74 years and just 9% of oldest adults ≥ 75 years were taking any kind of LLT. Low- or moderate-intensity statin therapy was most common, used in 9% of older adults, 7% of very-old adults, and 10% of younger adults, with no significant differences between age groups (*p* = 0.84). Pretreatment with high-intensity statin therapy was rare: only 2% of older adults and 2% of oldest adults were taking high-intensity statins at the time of STEMI presentation. Pretreatment with ezetimibe was also uncommon, with 2% of older adults and no very-old adults taking ezetimibe either in combination with statins or as monotherapy at the time of STEMI presentation, with no differences between age groups (*p* = 0.42). None of our high-risk/very-high-risk patients without prior ASCVD had been treated with PCSK9 inhibitors, although 2% of younger and older adults were taking fibrates at the time of STEMI presentation. Documented statin intolerance was uncommon, present only in 2% of older adults but not in younger or oldest adults (*p* = 0.31).

### 3.6 LLT use and LDL-C target achievement in very-high-risk patients with severe kidney disease (<30 mL/min/1.73 m^2^)

Patients with severe kidney disease, defined in both ESC/EAS Guideline years as those with eGFR <30 mL/min/1.73 m^2^, are always considered to be at very-high risk of ASCVD and are at higher risk of mortality than patients with CVD alone. While the use of statins or statin–ezetimibe combination therapy is recommended in this patient group, LLT implementation may be challenging due to the need for dose adaptations and/or dose-related adverse events. For this reason, we chose to extract patients with severe CKD and perform a sub-analysis on them separately. Only a very small number of patients in our study had prior severe kidney disease without dialysis at the time of STEMI presentation (3% adults < 65 years, *n* = 4; 3% older adults, *n* = 3; and 6% very-old adults, *n* = 7); however, results must be viewed with caution due to the sample size. Pretreatment with low-/moderate-intensity statins was observed in just three patients (one patient in each age group), while only one older adult had prior treatment with a high-intensity statin in combination with ezetimibe. Note that three patients in this cohort had a medical history of prior myocardial infarction with revascularization and/or ischemic stroke, and one had prior PAD, therefore establishing ASCVD. Median LDL-C was 107 mg/dL (IQR 70, 157) and median non-HDL was 121 mg/dL (IQR 78, 163) at admission for STEMI, with only three patients meeting the 2016 LDL-C target of < 70 mg/dL and no patient meeting the 2019 LDL-C target of < 55 mg/dL for very-high-risk patients.

### 3.7 LLT use and LDL-C target achievement in high-risk/very-high-risk patients without severe kidney disease (eGFR ≥ 30 mL/min/1.73 m^2^)

The ESC/EAS guidelines make specific mention with respect to LLT use in older patients with renal impairment, recommending slow up-titration of statins to meet risk-based LDL-C targets, especially as decreasing estimated glomerular filtration rate (eGFR) is clearly associated with increased CVD risk. Use of statins or statin/ezetimibe combination therapy is a 1A recommendation in patients at high- or very-high CVD risk with stage 3–5 kidney disease, yet the guidelines do urge caution with respect to dosing and potential dose-related adverse events. For this reason and as the use of statin therapies in patients with advanced CKD has been controversial, LLT prescription, use, and/or dosing may be more restrictive in patients with more severe renal impairment, thus influencing results. Therefore, we also undertook a sub-analysis of LLT uptake and LDL-C target achievement in high-risk/very-high-risk patients without severe renal disease (eGFR ≥ 30 mL/min/1.73 m^2^) by age (see Table 6).

**TABLE 6:** Lipid-lowering therapies and ESC/EAS^a^ lipid target achievement in patients without severe kidney disease (eGFR ≥ 30 mL/min/1.73^2^)^b^.

	Age <65	Age 65–74	Age ≥75	*p*-value
	*N* = 148	*N* = 101	*N* = 106	
Hyperlipidemia pretreated	31% (46)	34% (34)	35% (37)	0.74
Statin intensity				0.87
Low/moderate-intensity+	16% (24)	23% (23)	20% (21)	
High-intensity*	14% (20)	10% (10)	14% (15)	
Intensity unknown	1% (2)	1% (1)	1% (1)	
Pretreatment with ezetimibe	5% (7)	3% (3)	5% (5)	0.44
Unknown	0% (0)	1% (1)	0% (0)	
Pretreatment with other LLT				0.59
Fibrate	1% (2)	1% (1)	0% (0)	
PCSK9i	0%	0%	0%	
Known statin intolerance	1% (2)	3% (3)	2% (2)	0.67
LLT target achievement^a^				
Non-HDL				0.007
Non-HDL <85 mg/dL	15% (21)	29% (28)	32% (30)	
Non-HDL 85–99 mg/dL	9% (12)	14% (14)	12% (11)	
Non-HDL >99 mg/dL	76% (104)	57% (56)	56% (52)	
LDL				0.015
**LDL <55 mg/dL**	**6% (9)**	**18% (17)**	**24% (23)**	
**LDL 55–69 mg/dL**	**16% (22)**	**14% (14)**	**15% (15)**	
LDL 70–99 mg/dL	25% (35)	26% (25)	23% (22)	
LDL >99 mg/dL	53% (74)	42% (41)	38% (37)	

*High-intensity statins: atorvastatin ≥40 mg and rosuvastatin ≥20 mg. The parameters highlighted in bold are those required by ESC/EAS to determine LDL-C and non-HDL target attainment.

^+^low/moderate-intensity statins: atorvastatin < 20 mg, rosuvastatin < 20 mg, or all other statins/doses such as simvastatin, pravastatin, and fluvastatin.

^a^ ESC/EAS Guidelines, European Society of Cardiology (ESC)/European Atherosclerosis Society (EAS) Guidelines for the Management of Dyslipidemias (2016 and 2019).

^b^ eGFR, estimated glomerular filtration rate; LLT, lipid-lowering therapy; PCSK9i, proprotein convertase subtilisin/kexin type 9 inhibitor; non-HDL, non-high-density lipoprotein; LDL, low-density lipoprotein.

In our study, 97% of adults < 65 years, 97% of older adults aged 65–74 years, and 94% of very-old adults aged ≥ 75 years had eGFR ≥ 30 mL/min/1.73 m^2^ at presentation for STEMI. The median eGFR was 80 (69, 90) in younger adults, 68 (54, 82) in older adults, and 54 (43, 71) in very-old adults. Median LDL-C in this cohort was 104 mg/dL (IQR 76, 135), 85 mg/dL (63, 115), and 81 mg/dL (56, 121) in younger, older, and very-old adults, respectively. TC values were 177 mg/dL (148, 208) in younger adults, 156 mg/dL (130, 190) in older adults, and 152 (129, 201) in very-old adults at presentation for STEMI.

Despite being at high-/very-high-risk, just 31% of younger adults, 34% of older adults, and 35% of very-old adults in this population were pretreated with LLTs at the time of admission (*p* = 0.74). Again, pre-treatment with low-/moderate-intensity statin monotherapy was most common but was observed in just 16% of younger adults, 23% of older adults, and 20% of oldest adults (*p* = 0.87). High-intensity statin use upon admission for STEMI was seen in only 10% of older adults and 14% of both younger adults and very-old adults. Ezetimibe use, either alone or in combination therapy, was rare, observed in 3% of older adults and 5% of very-old adults. Known statin intolerance was low, recorded in 1%–3% of our high-risk/very-high-risk STEMI patients.

The corresponding achievement of LDL-C risk-based targets was also low, with 32% of older adults and 39% of very-old adults meeting the 2016 very-high and 2019 high-risk LDL-C target <70 mg/dL at the time of STEMI, with younger adults < 65 years having the poorest target achievement (22%) (*p* = 0.015). With respect to the more stringent 2019 LDL-C goal of < 55 mg/dL, just 6% of younger adults, 18% of older adults, and 24% of very-old adults met this target at the time of presentation for STEMI. Secondary non-HDL goals were also achieved in only a minority of patients. Just 43% of older adults and 44% of oldest adults met high-risk targets of < 100 mg/dL, with only 29% of older adults and 32% of very-old adults meeting the very-high-risk non-HDL target of < 85 mg/dL. Note that younger adults had the lowest achievement for both non-HDL targets (*p* = 0.007).

## 4 Discussion

### 4.1 Deficits in LLT implementation and LDL-C target achievement in older-/very-old adults with established ASCVD

The results of our study showed severe deficits in prior LLT use, with just 32%–36% of high-risk and very-high-risk patients on treatment for hyperlipidemia at the time of STEMI presentation, without significant differences by age group. Sub-optimal implementation of risk-based, guideline-directed therapies was observed in STEMI patients on prior treatment, both in younger adults and older adults 65–74 years and in very-old adults ≥75 years at the time of presentation for STEMI, although the severity of deficits in our study differed according to the presence or absence of prior ASCVD. As a consequence, the achievement of risk-based LDL-C targets was less than ideal in our older and very-old adult STEMI population. In our study, just over half (53%) of older and very-old ASCVD patients were pretreated with LLTs, predominantly with low-/moderate-dose statin monotherapy and ≤7% with combined ezetimibe, with just a 3% rate of statin intolerance reported. Approximately 12% and 17% of older and very-old patients with established ASCVD prior to STEMI did not survive to discharge.

A large body of evidence has underscored ESC/EAS guideline recommendations for statin use in secondary prevention in high-risk older patients > 65 years with established ASCVD in the same way as for younger patients (Boekholdt et al., 2014; Efficacy and safety of LDL, 2015; Catapano et al., 2016; Bach et al., 2019; Mach et al., 2020). Here, the causal role of LDL-C and the benefits of lipid-lowering therapy must be emphasized. A systematic review and meta-analysis of 21,292 older patients aged ≥ 75 years from statin, ezetimibe, and PCSK9i randomized control trials (RCTs) and the 24 Cholesterol Treatment Trialists (CTT) Collaboration studies demonstrated that LDL-C lowering significantly reduced the risk of major vascular events in older patients (≥75 years) by 26% per 1 mmol/L reduction in LDL cholesterol (RR 0.74 [95% CI 0.61–0.89; *p* = 0.0019]) with no statistically significant differences compared to that in patients <75 years (0.85 [0.73–0.91; p _interaction_ = 0.37]) (Gencer et al., 2020). Significant reductions were seen for all included composite endpoints, such as CV death, myocardial infarction, stroke, and coronary revascularization, regardless of age. Additionally, Gencer et al. (2020) found no offsetting safety concerns that would pose a barrier to treatment.

Another meta-analysis of statin use in older patients aged ≥ 65–82 years with established CVD showed a reduction in all-cause mortality, with an estimated relative risk reduction of 22% over 5 years with the use of statins (RR 0.78, 95% credible interval CI 0.65–0.89). Moreover, a reduction of 30% was also observed in coronary heart disease mortality (RR 0.70; 95% CI 0.53–0.83); non-fatal myocardial infarction, 26% (RR 0.74; 95% CI 0.6–0.89); need for revascularization, 30% (RR 0.7; 95% CI 0.53–0.83); and occurrence of stroke, 25% (RR 0.75; 95% CI 0.56–0.94). A posterior median estimate of NNT to save 1 life was 28 (95% CI 15–56) (Afilalo et al., 2008). In our study, however, 47% of older and very-old adults with established ASCVD were not taking any LLTs at the time of admission for STEMI, suggesting either potential adherence or intolerance issues or deficits in follow-up care delivery after their first ASCVD event or diagnosis. Note that 71% and 79% of older and very-old ASCVD were taking and prescribed medications for comorbidity hypertension and thus were managed by a healthcare provider, yet risk-based LLT was not implemented in these patients despite low reported statin intolerance (3, 2%).

Of particular note is the relatively low incidence of statin intolerance (SI) observed among our real-world STEMI patients, in contrast to findings from a large, ESC/EAS meta-analysis of 176 studies in 4 million patients worldwide, which showed a 9.1% [95% CI, 8%–10%] pooled prevalence of SI, regardless of statin type, and a 5.9% [4.0%–7.0%] SI incidence when using EAS diagnostic criteria (Bytyçi et al., 2022). The authors also noted that SI incidence was significantly lower in RCTs compared to cohort studies [4.9% (4.0%–6.0%) vs. 17% (14%–19%)], an observation not aligned with our results. Especially interesting, however, was the 13% [95% CI, 2.0%–24%] SI incidence described in secondary prevention acute coronary syndrome patients, compared to the 2%–3% seen among our patients with prior ASCVD at presentation for STEMI. In a meta-regression analysis, Bytyçi et al. (2022) observed that age as a continuous variable was significantly associated with a higher SI risk [odds ratio OR 1.33, 95% CI 1.25–1.41, *p* = 0.04], yet in our study, no significant differences in SI were observed between age groups, perhaps explained by our comparatively small sample size.

According to the Cholesterol Treatment Trialists’ Collaboration, more-intensive statin regimens produce a highly significant 15% further reduction in major vascular events compared to less intensive regimens, primarily through significant reductions in coronary death and non-fatal myocardial infarction (Cholesterol Treatment Trialists CTT Collaboration et al., 2010). The SAGE study (Study Assessing Goals in the Elderly) study also showed an association between high-intensity statin therapy and greater reductions in LDL-C, the occurrence of major acute cardiovascular events, and death in patients aged 65–85 years of age when compared to the use of moderate-intensity statin therapy (Deedwania et al., 2007).

However, in our study, 34% and 29% of older and very-old ASCVD patients were treated with low/moderate dose statin therapy at the time of STEMI, respectively, and just 17% and 20% of older/very-old patients were treated with high-intensity statin therapy, despite guideline recommendations encouraging up-titration of statins to meet risk-based LDL-C guideline targets (2016) or prescription of a high-intensity statin titrated to the highest tolerated dose (2019), with the addition of ezetimibe if targets are unmet (2019) in very-high-risk populations. Ezetimibe was only used in 5%–7% of our older and very-old ASCVD patients at the time of admission for STEMI. Our findings align with those of a US study of high-intensity statin and non-statin LLT use in older patients ≥ 75 years with ASCVD. In that study, 49.3% were taking any statin, with 16.6% taking a high-intensity statin, 32.7% taking a low-/moderate-intensity statin, 2.4% on ezetimibe, and a rare use of PCSK9is (0.24%) (Nanna et al., 2023). Although we cannot confirm in our retrospective study whether the observed doses were actually those most tolerated, our findings still highlight deficits in the intensification of statin therapy and/or in the expansion of therapy with ezetimibe in the majority of our older and very-old patients with established ASCVD. A secondary analysis of the IMPROVE-IT study, an RCT examining combined statin–ezetimibe therapy versus statin monotherapy in ACS patients, demonstrated that the greatest absolute risk reduction was observed among patients aged ≥75 years. Addition of ezetimibe to statin treatment was not associated with a significant increase in safety issues among the oldest patients (Bach et al., 2019).

It is important to note that none of our STEMI patients were on prior treatment with PCSK9 inhibitors, despite the 1A recommendation for their use in secondary prevention patients not meeting LDL-C targets at maximally tolerated doses of statin–ezetimibe therapy. Although PCSK9is were introduced to the Austrian market in 2016, lack of use or prescription may potentially be attributed to high costs, initially restrictive prescribing policies by social insurance carriers, or concerns about weaker evidence regarding their use in older populations underrepresented in market-entry RCTs.

Sub-optimal achievement of LDL-C targets was observed in our secondary prevention patients, aligning with the described LLT implementation deficits: only 43% of older adults and 49% of very-old adults with established ASCVD met 2016 LDL-C targets <70 mg/dL, and just 22% of older adults and 30% of very-old adults met stricter 2019 LDL-C targets <55 mg/dL at the time of presentation for STEMI. Several large European registries and observational studies, such as Da Vinci, EUROASPIRE-V, and SANTORINI studies, describe gaps between guideline recommendations and actual clinical practice (De Backer et al., 2019; Ray et al., 2021; Gouni-Berthold et al., 2022; Ray et al., 2023). Although not differentiated by age, the EU-wide observational Da Vinci study of LLT use and LDL-C target achievement in 5,888 primary and secondary care patients noted that just 35% of the patients with established ASCVD (*n* = 2,794) taking moderate-intensity statin monotherapy met the 2016 targets and 16% met the 2019 targets. In contrast, 45% of ASCVD Da Vinci patients taking high-intensity statin monotherapy met 2016 and 22% met 2019 LDL-C goals, respectively, highlighting persisting deficits in LDL-C goal attainment even in those patients prescribed and taking LLTs (Ray et al., 2021). In those Da Vinci ASCVD patients taking statin–ezetimibe combination therapy, 54% met 2016 LDL-C targets and 21% achieved more stringent 2019 goals. The mean age of ASCVD patients was 68 years (SD 10), thus roughly corresponding to the age of our older and younger patient populations. As in the Da Vinci study, we observed some discrepancy between the 53% of older adults and very-old adults taking LLTs at the time of STEMI and the respective rates of LDL-C attainment. Interesting to note were higher percentages of very-old adults achieving stricter LDL-C < 55 mg/dL targets than older adults aged 65–74, and there was less of a treatment discrepancy in LDL-C target attainment in the oldest group. Observations from a Danish nationwide cohort study (*n* = 82,958) describe large patient-to-patient variability in LDL-C responses to statin treatment, and the authors observed that initiation of low–moderate-intensity statins was associated with greater reduction in LDL-C levels in oldest patients (age > 75) than in younger patients, both in primary and secondary prevention patients (Corn et al., 2023), offering a potential explanation for the higher treatment response in our oldest ASCVD patients. Older adults had higher plasma concentrations than younger adults, which authors suggested may be linked to greater bioavailability of statins and greater drug absorption in older patients, or to age-related changes in hepatic function, leading to increased statin exposure, or to impairment in renal function potentially affecting statin concentrations (Corn et al., 2023).

Relevant to our study were the results of the EU-wide Santorini study, which focused on LLT implementation and achievement of 2019 guideline LDL-C targets among high- and very-high-risk patients in diverse primary and secondary care settings in 14 European countries (*n* = 9,044), including Austria (*n* = 310), in 2020–2021 (Ray et al., 2023). Among the 9,044 patients enrolled in the Santorini study, the majority (73.3%) did not achieve 2019 LDL-C goals, and the median LDL-C was out of target, both in high-risk (93 mg/dL, 2.4 mmol/L) and very-high-risk patients (78 mg/dL, 2.0 mmol/L). A total of 6,954 patients (76.9%) had prior ASCVD, thus classifying them as very high-risk. Among Santorini ASCVD patients, 21.4% were not taking any LLTs at baseline, 53% were taking statin monotherapy, and just 25.6% were taking combination LLTs (Ray et al., 2023). One key message in the study was that LDL-C targets were not attained in the vast majority of very-high-risk patients, even in those using high-intensity statin monotherapy, and the authors concluded that combination therapies proven to effectively lower LDL-C levels still have not found widespread use in Europe. This finding mirrors the results of our study. The finding that 1,094 (15.7%) patients with ASCVD were incorrectly classified by their physicians as high-risk instead of very-high risk was alarming as well, indicating an underestimation of patient risk and perhaps contributing to sub-optimal LLT implementation with resultant LDL-C not at target levels (Ray et al., 2023).

In the Austrian Santorini cohort (*n* = 310), 26.1% of patients were not taking any LLT, 48.1% were on statin monotherapy, and 25.8% were taking combination therapies at baseline (Ray et al., 2023). The resulting out-of-target median LDL-C levels (78.1 mg/dL, 2.02 mmol/L) demonstrated sub-optimal LLT implementation among high- and very-high-risk patients (Ray et al., 2023). When compared to the total Santorini study population and Austrian sub-cohort, our real-world STEMI population had even higher LDL-C medians (82–103 mg/dL) and inversely lower rates of 2019 LDL-C target achievement in just 15%–16% of high-risk and 6%–23% of very-high-risk patients across all age groups. Severe deficits in LLT implementation were observed among our patients, as 64%–68% of high-/very-high-risk patients were not taking any LLT at presentation for STEMI, 16%–20% were taking low-/moderate-intensity statins, and just 11%–13% were taking a high-intensity statin with only 4%–5% on combination therapy, more severe deficits than observed among participants in the Santorini study, without significant differences between age groups.

### 4.2 Deficits in LLT implementation and LDL-C target achievement in older adults (aged 65–74) without established ASCVD

With respect to primary prevention, the 2019 guidelines recommend a risk-based approach for utilization of statins for older patients ≤ 75 years (1A recommendation) and consideration for their use in high-/very-high-risk patients >75 years (IIb/B), while the 2016 guidelines, also in place during our study period, make a general IIa/B recommendation for “consideration of their use in older adults free of CVD, particularly in the presence of risk factors hypertension, smoking, diabetes and dyslipidemia” (Catapano et al., 2016; Mach et al., 2020). In our study, just 16% of older adults aged 65–74 years without established ASCVD but with high-/very-high-risk criteria were taking LLTs for primary prevention at the time of STEMI. Nine percent of patients were taking low-/moderate-intensity statin therapy, with 2% on high-intensity statin treatment at the time of STEMI, although the statin intensity was unknown. Ezetimibe use was low at 2%. With respect to LDL-C target attainment, 48% of older patients met 2016 high-risk LDL-C targets of < 100 mg/dL, while 17% attained the 2016 very-high-risk/2019 high-risk LDL-C target of < 70 mg/dL. Just 10% of patients aged 65–74 years met the 2019 very-high-risk LDL-C target <55 mg/dL. A 2% statin intolerance was reported in this age group.

Note that 66% of these patients were on treatment for hypertension and 51% were treated for the comorbidity DM, suggesting potential care-delivery deficits with respect to low rates of risk-based LLT implementation, especially following the more direct 2019 guideline recommendations. However, with respect to the 2016 ESC/EAS IIa/B recommendation, the decision not to implement LLT may have often been a conscious one, especially amid the debate regarding the time to benefit of statin treatment in primary prevention in this age group and uncertainty about the effects of statins in older adults (Yourman et al., 2021) In a US meta-analysis evaluating the time to benefit of statin use in primary prevention, including 60,383 patients aged 50–75 years, Yourman et al. (2021) concluded that treating 100 adults without established CV disease in this age group with a statin for 2.5 years would likely yield prevention of one MACE in one adult. In contrast, results of a British meta-analysis in 70,388 patients concluded that statins in primary prevention improve survival and reduce the risk of major CV and cerebrovascular events in people without established CVD, with equal treatment benefits across a range of clinically defined groups (men/women, older adults > 65 years, and those with DM) (Brugts et al., 2009).

### 4.3 LLT implementation and LDL-C target achievement in older adults (aged ≥ 75) without established ASCVD

The use of statin therapy for primary prevention in oldest adults > 75 years is contentious, especially due to multi-morbidities, frailty, polypharmacy, altered pharmacokinetics and pharmacodynamics, and safety concerns with respect to drug-related adverse events or drug–drug interactions, potentially outweighing treatment benefits. In this context, both 2016 and 2019 guidelines are careful with recommendations for initiation of statin therapy for primary prevention in oldest patients >75 years with high-/very-high-risk profiles, which “may be considered” (IIb/B) (Catapano et al., 2016; Mach et al., 2020). In our study, 9% of oldest adults with high-risk/very-high-risk profiles were on prior treatment with statin at the time of presentation for STEMI, with 7% taking low-/moderate-intensity therapy and 2% taking high-intensity therapy. No patient was using ezetimibe alone or as a combination therapy. No statin intolerance was reported. This age group had the highest rates of renal insufficiency (70%) and prior heart failure (14%), potentially influencing decisions to initiate LLT. Approximately 20% of patients in this age group had LDL-C values < 70 mg/dL and 10% had LDL-C values < 55 mg/dL at the time of presentation for STEMI.

The literature offers mixed evidence regarding the appropriateness, use, and benefit of statin therapy in oldest adults without overt ASCVD. A US Veterans observational study of 326,981 predominantly male patients ≥ 75 years free of ASCVD at baseline showed that initiation of statin therapy was significantly associated with a lower risk of all-cause and CV death (Orkaby et al., 2020). A French study evaluating the new use of statins in 7,284 patients aged ≥ 75 years to lower the risk of acute coronary syndrome or all-cause death with a 4.7-year follow-up showed that cumulative use of statins was associated with a lower risk of outcomes in primary prevention patients with modifiable risk factors as well as in secondary prevention patients, but not in primary prevention patients without modifiable risk factors (Bezin et al., 2019). However, in a meta-analysis of individual participant data from 28 RCTs, the CTT collaboration authors concluded that while statin therapy produces significant reductions in major vascular events irrespective of age, there was a less direct benefit in patients > 75 years without evidence of prior occlusive vascular disease (Gencer et al., 2020).

### 4.4 LLT implementation and LDL-C target attainment in the context of renal impairment

Both the 2016 and 2019 guidelines make unequivocal recommendations for statin or combined statin–ezetimibe use in patients with CKD stages 3–5 to address concurrent high ASCVD risk, yet the guidelines urge caution when dosing due to increased blood concentrations of compounds with the potential for drug-related adverse events in this population (Catapano et al., 2016; Mach et al., 2020). Among the small cohort of patients with severe CKD (eGFR < 30 mL/min/1.73 m^2^) in our study, only 3 out of 14 (21%) were on treatment with a low-/moderate-intensity statin and only 1 was treated with high-intensity statin–ezetimibe combination therapy at the time of STEMI. Corresponding LDL-C target achievement was low, with 3 patients meeting the <70 mg/dL LDL-C targets and none attaining stricter < 55 mg/dl LDL-C goals. However, in a meta-analysis examining the effect of renal function on LDL cholesterol lowering in patients with severe kidney disease, the Cholesterol Treatment Trialists’ Collaboration determined that statin-based therapy reduced the risk of a first major vascular event by 21% (RR 0.79, 95% CI 0.77–0.81; *p* < 0.0001) per mmol/L on LDL-C reduction (Cholesterol Treatment Trialists' CTT Collaboration et al., 2016). Reductions in LDL-C, however, became smaller with more advanced CKD. In parallel, reductions in major vascular events observed with the use of statin-based therapies became smaller as eGFR declined, with little or no benefit derived in patients on dialysis. The authors concluded that in patients with severe CKD, statin-based regimens should be selected to maximize absolute LDL-C reduction to attain maximal therapeutic benefits (Cholesterol Treatment Trialists' CTT Collaboration et al., 2016).

As severe renal impairment may influence prescribing, uptake, and dosing of statins and is often cited as the reason for drug-related adverse events, we removed patients with severe renal impairment (eGFR < 30 mL/min/1.73 m^2^) in a sub-analysis to determine potential effects on LLT implementation and corresponding LDL-C target achievement results in the remaining cohort, especially as prior renal insufficiency was significantly prevalent in 29% older and 50% very-old adults, both with and without prior ASCVD, compared to younger adults. However, after removing these patients, similar rates of pretreatment LLT were observed between age groups in 31%, 34%, and 35% of younger, older, and very-old adults, respectively. Rates of low-/moderate-intensity statin use were also comparable between age groups (16%, 23%, and 20%, respectively), as was the less-common use of high-intensity statins (14%, 10%, and 14%) and the rare use of ezetimibe (5%, 3%, and 5%). Significant differences in LDL-C target attainment by age were observed in this cohort, with the highest attainment of LDL-C targets < 70 mg/dL found among older (32%) and oldest adults (39%) and the achievement of stricter LDL-C targets < 55 mg/dL observed in just 18% of older and 24% of very-old adults. These results mirror the findings of our total cohort. Note that these findings allow no justification for missing, low, or non-optimized LLT implementation, as the guidelines make a 1A recommendation for statin or combined statin–ezetimibe use in patients with stages 3–5 CKD at high- or very-high CVD risk (Mach et al., 2020).

### 4.5 Healthcare delivery deficits

Sub-optimal implementation of guideline-directed, risk-based LLTs was seen in our study, especially among older and very-old adults with established ASCVD (secondary prevention) as well as in older adults < 75 years without prior ASCVD but with a high-/very-high-risk profile (primary prevention) at the time of presentation for STEMI.

Severe healthcare delivery deficits were observed among secondary prevention patients across all age groups. With respect to older and very-old adults with established ASCVD, most were only taking statin monotherapy and were treated with low-/moderate-intensity statins (34, 29%), despite the low achievement of LDL-C targets, meaning that dose intensity had not been optimized in many patients on prior treatment. Few of our older-/very-old very-high-risk patients were taking high-intensity statins (17, 20%), and fewer were using ezetimibe therapy (5%–7%), despite the evidence of its efficacy and recommendation for use. The percentage of patients with documented statin intolerance was low (3%) in our older/very-old populations; thus, the lack of therapy intensification cannot solely be attributed to statin intolerance. Our findings align with those of several studies describing the underuse of statins in older ASCVD populations (Ko et al., 2004).

Especially worrisome is the finding that 47% of our very-high-risk older/very-old adults with established ASCVD were not on any LLTs at the time of STEMI, despite their demonstrated efficacy in preventing subsequent events. Considering that 71% of older and 79% of very-old ASCVD patients were prescribed and taking medications for the comorbidity hypertension and were thus managed by a healthcare provider, the lack of LLT use in large percentages of these patients highlights a severe deficit in follow-up care in these very-high-risk patients after an initial ASCVD event or diagnosis. Coupling the prescription of hypertension medications and/or the prescription of medications for other comorbidities represents a strategy for improving LLT uptake. As patients in Austria are required to physically pick up prescriptions from their general practitioners or internists, the prescription of an LLT at the time of prescription for other conditions was either overlooked, adherence issues/side-effects were not addressed, or a very high risk was not recognized by a healthcare provider. In primary prevention among high-/very-high-risk patients aged 65–74 years, single digit rates of statin use were reported with low attainment of risk-based LDL-C targets, although here poor LLT implementation may be attributed to weaker 2016 guideline recommendations. However, the 2019 guidelines issued a 1A recommendation for their use in high-/very-high-risk patients aged ≤ 75. Therefore, our findings show deficits in uptake. Again, 66% of patients were concomitantly treated with medications for hypertension and 51% for diabetes mellitus, suggesting that healthcare providers were not recognizing or appropriately managing risk.

The European Society of Cardiology provides evidence-based risk prediction tools and resources for physicians and allied health professionals to align patient characteristics, clinical signs, and laboratory tests with accurate, objective prediction of risk to support appropriate treatment strategies, improve clinical outcomes, and avoid both overtreatment of low-risk individuals and undertreatment of those with higher risk (Rossello et al., 2019). Validated risk prediction tools such as the Systematic Coronary Risk Evaluation (SCORE2) model and SCORE-OP (for older persons) may be used with patients to discuss risks, tailor patient counseling, encourage adherence to medications and lifestyle changes, and facilitate shared treatment decisions (SCORE2-OP working group and ESC Cardiovascular risk collaboration et al., 2021; Rossello et al., 2019; SCORE2 working group and ESC Cardiovascular risk collaboration et al., 2021). However, some obstacles to its routine implementation in daily practice have been described, such as lack of time or the perceived simplicity of the algorithm in contrast to patient complexity, which causes resistance among some physicians (Rossello et al., 2019). Risk prediction tools allow healthcare providers to more accurately gauge risk and tailor LLT to meet risk-based LDL-C goals in ASCVD populations. Greater uptake of these tools has the potential to remedy deficits in LLT implementation and LDL-C target achievement observed in STEMI populations across all age groups.

Poor patient adherence and/or diminishing uptake of LLTs over time in older populations, often in conjunction with LLT side effects, such as statin-related muscle pain, have been reported in the literature (Cheeley et al., 2022). The ESC/EAS guidelines recommend addressing any potential statin side effects with patients and providing healthcare providers with strategies for gradual dose up-titration, the addition of ezetimibe, and/or potentially PCSK9is to achieve LDL-C targets (Mach et al., 2020). If a conscious decision to deprescribe statins was made due to polypharmacy, potential adverse reactions, and/or concerns about treatment complications, then effective alternative therapies, for example, with PCSK9 inhibitors, were not initiated in our patients, also highlighting a potential care-delivery deficit. An Italian population-based study of nearly 30,000 older patients with mean age 76.5 years described the consequence of deprescribing statins in older patients with polypharmacy, associating statin depresciption with an increase in the long-term risk of fatal and non-fatal CV outcomes, especially in high-risk patients (Rea et al., 2021).

Non-adherence to evidence-based therapies for CVD is multifactorial and has been attributed to sociodemographic, psychological, economic, and clinical factors as well as the complexity of treatment regimens, polypharmacy, and pill burden, especially common in older patients (Bramlage et al., 2017; Tamargo et al., 2022). Use of a polypill is one option to address sub-optimal drug adherence and has been shown in multiple studies to significantly improve adherence to long-term regimens (Bramlage et al., 2017). The SAGE (secondary prevention of cardiovascular disease in the elderly) demonstrated significant improvements to CVD medication adherence in older adults ≥ 65 years with a corresponding reduction in major adverse CV events 6 months post-MI through the use of a polypill compared to individual medication doses (Castellano et al., 2022). A German retrospective study of statin and ezetimibe prescribing practices in over 300,000 CVD patients also described higher LLT adherence rates when using a fixed dose statin–ezetimibe polypill versus individual pill intake, noting cardiologists were more likely to prescribe a polypill with high-intensity statins than GPs, who tended to prescribe low-to-moderate-intensity statin monotherapy, with low rates of add-on ezetimibe therapy prescription (Katzmann et al., 2022).

Mobile health (mHealth) tools offer another new modality for providing patient education and adherence support using mobile devices, such as mobile phones and other personal monitoring devices, falling loosely under the rubric of telemedicine (Gandapur et al., 2016). Automated SMS reminders, alarms, and voice messaging have been shown to increase adherence to CV medicines, with some studies describing higher percentages of correct doses, doses taken on time, and improved cumulative adherence (Park et al., 2014; Vollmer et al., 2014; Wald et al., 2014), although study designs and sizes varied and did not specifically focus on use of mHealth tools among older adults. Note that despite the use of statins, LDL-C targets are not always achieved in all patients. A large Australian population study of 61,000 patients retrospectively examined LDL-C goal achievement among all risk groups and found that only 36% of patients on statin therapy actually met therapeutic targets (Talic et al., 2022). These findings only partially align with the observations of our study. Although 53% of our older patients with ASCVD were on treatment, just 43% met 2016 and 22% met 2019 LDL-C targets. The discrepancy between LLT use and LDL-C target achievement was less pronounced among very-old patients with established ASCVD (53% LLT use versus 49% and 30% achievement by guideline year). Our findings emphasize the importance of LDL-C follow-up measurement and the importance of therapy optimization if LDL-C targets are unmet. Follow-up control cannot be left to chance but requires policies to ensure guideline-directed therapy implementation and optimization to reduce potential future adverse CV events.

When current LLTs are inadequate or not well-tolerated, emerging alternative classes of drugs have been shown to lower LDL-C, such as the small interfering RNA injectable, inclisiran, as an alternative to PCSK9 inhibitors, or the ATP citrate-lyase inhibitor, bempedoic acid, for statin-intolerant patients, and may offer benefit, although robust data for their use in older and very-old adults are still needed (Mach et al., 2020).

Identification and follow-up of very-high-risk patients are essential not only for the control of LDL-C but also as an opportunity for management of other ASCVD risk factors, such as overweight/obesity and smoking. In our study, more than three-quarters of older STEMI patients were overweight/obese and 32% of older patients with a very-high-risk profile were actively smoking at the time of STEMI presentation, demonstrating the need for more rigorous lifestyle risk factor management in these patients.

The ESC has urged improvement in preventative care, especially through the use of secondary prevention programs (inpatient, outpatient, and long-term interventions), cardiac rehabilitation, and multidisciplinary preventive services in the community (Piepoli et al., 2016). Yet the ESC estimates that only one-third to one-half of eligible patients are referred to appropriate prevention programs, identifying barriers at the patient, healthcare provider, and healthcare system levels. At the patient level, hurdles include not receiving or understanding information from healthcare providers, lack of social support, poor psychological wellbeing, lack of access to programs, and competing work and family commitments (Piepoli et al., 2016). At the healthcare provider level, educational gaps in detailed preventive care knowledge among cardiologists, GPs, and allied healthcare specialists, a shift from longer hospital stays to less expensive outpatient treatment, leaving a limited amount of time and resources for education, inappropriate risk stratification, a lack of or inadequate post-discharge strategies to support patients, and suboptimal communication between acute care and primary care healthcare providers all contribute to inadequate referral or enrollment of patients to prevention programs (Piepoli et al., 2016). Limiting factors at the healthcare system level include a lack of available prevention centers or rehabilitation programs for all regions, a lack of minimum standards for the quality and delivery of secondary prevention programs, the need for accountability measures such as referral performance and evaluation of appropriate prescriptions of evidence-based medications at the system level, as well as the need for structured, multidisciplinary care pathway plans to be used by health services to guide the referral and management of patients qualifying for risk-based (secondary) prevention programs (Piepoli et al., 2016). Life expectancy in high-income countries for patients aged 75 is expected to be at least 10 years (Gencer et al., 2020); therefore, adequate risk control and risk reduction are essential for longevity, maintenance of functional status, and quality of life, both in the interest of patients and with respect to healthcare system costs and burden.

### 4.6 Limitations

Our findings should be interpreted in the context of several limitations. The main limitation was the single-center, retrospective design of our study. Therefore, our results may not reflect LLT implementation or LDL-C target achievement in older and very-old adult high-risk/very-high-risk populations in other EU regions. Yet our study serves as a healthcare delivery quality indicator in our region, and the sub-optimal implementation of guideline-directed, risk-based LLTs and poor LDL-C target achievement, especially among older/very-old patients with established ASCVD, observed in our study is a finding potentially applicable in other regions. An important limitation to be highlighted is that 54 patients or 5.6% of the entire STEMI patient population were excluded as no LDL-C and/or TC was available, despite clear guideline recommendations for clinical risk assessment during STEMI hospitalization. Lack of testing may have been caused by either patient death, staff oversight, or patient transfer out of our clinic prior to testing, partially explained in 2020 by disrupted care delivery processes at the start of the SARS-CoV-2 pandemic.

Our study had other limitations to report. Although lipoprotein-B measurement is suggested in the guidelines, this parameter is not routinely measured in STEMI patients at our hospital, however, both ESC/EAS guidelines determined risk and treatment targets using LDL-C and TC, which were the parameters used in our study. The ESC/EAS guidelines offer a scoring system for calculation of risk for 10-year fatal CVD. We did not use the SCORE calculator to solely classify patients as high/very-high risk in our retrospective study, as we could not confirm whether blood pressure measurements at the presentation for STEMI required for the SCORE calculation were performed in a harmonized way. Therefore, the actual number of high-/very-high-risk patients may be underestimated. The newer SCORE-OP, a specific calculator for determining risk in older persons, was not yet published at the time of our study and, therefore, was not used. Another potential group of very-high-risk patients not specifically captured in our study concerns those with FH, although FH patients with confirmed ASCVD were included by default. However, FH and only one major risk factor may not have been recognized as very-high risk, again possibly resulting in an underestimation of the total number of very-high-risk patients. Another limitation concerns the results of the sub-analysis in patients with severe CKD, which must be viewed with caution due to the very small sample size.

A retrospective study cannot confirm a causative effect of LDL-C exceeding guideline-recommended target levels with the presentation for STEMI, despite the implication of LDL-C in the development of ASCVD. However, as widely documented, LDL-C reductions correlate to reductions in all-cause mortality and occurrence of major adverse cardiovascular events, such as STEMI. Our study therefore only seeks to provide insights into real-world clinical practice with respect to LLT implementation and current lipid profiles in older/very-old high-/very-high-risk patients at the time of presentation for STEMI.

## 5 Conclusion

Our findings demonstrate critical shortcomings in real-world clinical practice with respect to implementation and optimization of guideline-directed, risk-based LLTs among high/very-high-risk older and very-old adults at the time of presentation for STEMI, with corresponding low achievement guideline-recommended LDL-C targets. Missing or non-optimized LLT implementation was observed in many ASCVD patients, indicating care-delivery deficits in therapy optimization, especially as less than half of older and very-old adults met the 2016 LDL-C target (43, 49%) and less than one-third attained stricter 2019 LDL-C targets (22, 30%).

In primary prevention, prior treatment with LLTs and LDL-C target achievement must be examined in the context of guideline revisions and diverging recommendations for age groups < 75 years and ≥ 75 years. In high-risk/very-high-risk older patients (65–74 years), with the 2019 1A recommendation for statin or statin–ezetimibe combination therapy, 16% prior treatment with statin therapy and 2% pretreatment with ezetimibe were alarmingly low, revealing potential shortcomings in risk identification and LLT initiation by healthcare providers. The percentage of risk-based LDL-C achievement among older adults 65–74 years differed according to level of risk and guideline year, with 48% of patients meeting LDL-C targets for high-risk patients (2016 guidelines), 17% meeting very-high-risk (2016)/high-risk (2019) LDL-C targets, and 10% meeting 2019 very-high-risk LDL-C targets.

Among oldest adults ≥ 75 years without established ASCVD but with a high-risk/very-high-risk profile, prior LLT with low-/moderate-dose statin monotherapy was observed in just 9% of patients, likely due to weaker IIb/B guideline recommendations and amid contentious debate regarding LLT initiation in the context of multi-morbidity, frailty, polypharmacy, and concern for drug-related adverse events, although mounting evidence has demonstrated that LDL-C lowering significantly reduces the risk of major vascular events in older patients (≥ 75 years) without offsetting safety concerns, which would pose a barrier to treatment.

## Data Availability

The raw data supporting the conclusion of this article will be made available by the authors, without undue reservation.
